# Effect of (+)-dehydrofukinone on GABA_A_ receptors and stress response in fish model

**DOI:** 10.1590/1414-431X20154872

**Published:** 2015-11-27

**Authors:** Q.I. Garlet, L.C. Pires, D.T. Silva, S. Spall, L.T. Gressler, M.E. Bürger, B. Baldisserotto, B.M. Heinzmann

**Affiliations:** 1 Programa de Pós-Graduação em Farmacologia, Universidade Federal de Santa Maria, Santa Maria, RS, Brasil; 2 Curso de Farmácia, Universidade Federal de Santa Maria, Santa Maria, RS, Brasil; 3 Programa de Pós-Graduação em Engenharia Florestal, Universidade Federal de Santa Maria, Santa Maria, RS, Brasil

**Keywords:** Sedation, Anesthesia, Rhamdia quelen, Nectandra grandiflora, Cortisol

## Abstract

(+)-Dehydrofukinone (DHF) is a major component of the essential oil of *Nectandra grandiflora* (Lauraceae), and exerts a depressant effect on the central nervous system of fish. However, the neuronal mechanism underlying DHF action remains unknown. This study aimed to investigate the action of DHF on GABA_A_ receptors using a silver catfish (*Rhamdia quelen*) model. Additionally, we investigated the effect of DHF exposure on stress-induced cortisol modulation. Chemical identification was performed using gas chromatography-mass spectrometry and purity was evaluated using gas chromatography with a flame ionization detector. To an aquarium, we applied between 2.5 and 50 mg/L DHF diluted in ethanol, in combination with 42.7 mg/L diazepam. DHF within the range of 10-20 mg/L acted collaboratively in combination with diazepam, but the sedative action of DHF was reversed by 3 mg/L flumazenil. Additionally, fish exposed for 24 h to 2.5-20 mg/L DHF showed no side effects and there was sustained sedation during the first 12 h of drug exposure with 10-20 mg/L DHF. DHF pretreatment did not increase plasma cortisol levels in fish subjected to a stress protocol. Moreover, the stress-induced cortisol peak was absent following pretreatment with 20 mg/L DHF. DHF proved to be a relatively safe sedative or anesthetic, which interacts with GABAergic and cortisol pathways in fish.

## Introduction

Within pharmacology, there has been a continuous search for new bioactive molecules, and plants with biological activity have been a focus for drug discovery (1). In particular, essential oils (EO) are considered to be a profitable source of new substances with biological activities (2,3). For example, linalool, a plant-derived monoterpene alcohol and a constituent of many EOs, acts as an anesthetic and sedative in silver catfish (4) and, together with linalyl acetate, promotes anxiolytic-like effects in mice (5). Such effects have also been demonstrated with myrtenol via a GABAergic mechanism (6). Spathulenol, a sesquiterpene component from the EO of *Aloysia gratissima*, was patented as a fish anesthetic due to its strong depressant effects on neuronal activity (7). Therefore, EOs can be a productive source of new molecules, particularly those exhibiting depressant effects on the central nervous system (CNS).

CNS depressants, such as sedatives and anesthetics, are incredibly important in human and animal health (8,9). Anesthetics are applied in various situations, such as for respiratory and pain control, loss of consciousness or local sensation in surgical procedures, and to facilitate animal handling by veterinarians (10,11). However, significant side effects are associated with the use of synthetic compounds currently available as anesthetics for aquatic animals, such as tricaine methanesulfonate (MS-222), benzocaine, and metomidate. These side effects include mucus loss, depression of cardiovascular and respiratory function, and immunosuppressive effects (10,11).

Recently, our research group has studied EOs and their isolated compounds, focusing on the development of CNS depressants with fewer side effects (7,8,11). To understand how sedation and anesthesia occur, several studies have investigated the neuronal pathways targeted by EOs and their derivatives (5,6). The GABAergic system acts to reduce neuronal transmission. As such, this system is implicated in the action of several sedatives and anesthetic drugs (5,6,12). In particular, GABA_A_ receptors play a prominent role. These receptors are widely expressed among vertebrates, from fish to mammals, and have been detected in zebrafish brains (13). Several studies have reported the action of EOs on GABA_A_ receptors in mice or fish models (2,4,5,6). Fish, as experimental animals, have proved to be a promising new approach in drug investigation, including natural substances with depressant effects (4,7,8,14). Indeed, silver catfish (*Rhamdia quelen*) provide a robust model, which is currently used by our group to screen EOs and their components for sedative and anesthetic activity (4,7,8,11,14).

Brazilian flora includes several species with EOs that have not yet been studied. Prominent among these species is a native endemic tree from the Brazilian Atlantic forest, *Nectandra grandiflora* Ness, ordinarily known as “canela-amarela” (15). The major constituent of its EO was identified as (+)-dehydrofukinone (2(3H)-naphthalenone, 4,4a,5,6,7,8-hexahydro-a,5-dimethyl-3-(1-methylethylidene)-,(4ar-cis)) (DHF) (16). This eremophylane-type sesquiterpene, also known as dihydrokaranone, was previously isolated from *Senecio*species, *Cacalia hastata* methanolic extract, and agarwood (17). Our research group has patented DHF in Brazil as an anesthetic for aquatic animals, using silver catfish as an experimental model. The sedative and anesthetic effects of DHF were first reported by Heinzmann et al. (18) at 9 and 50 mg/L, respectively. The anesthetic properties of DHF are equivalent to eugenol, a well-known anesthetic derived from clove oil. However, it has been demonstrated that DHF can be used at higher concentrations than eugenol without side effects or mortality (8,18). Furthermore, sensory analysis of eugenol has indicated that this compound modifies the flavor of fish fillets and therefore it is not appropriate for use as an anesthetic for fish destined for human consumption (8). DHF is also more effective as an anesthetic in fish models than spathulenol (7,18).

The present study aimed to investigate the role of GABA_A_ receptors in CNS depression caused by DHF isolated from the EO of *N. grandiflora,*using silver catfish as an experimental model. Therefore, we used a classic benzodiazepine drug association protocol and a GABA_A_ benzodiazepine receptor site antagonist, flumazenil, for CNS depression reversal. Considering potential harm caused by anesthetics to animals in aquaculture, we evaluated the action of DHF on plasma cortisol levels, and studied whether fish undergoing long DHF exposure experienced side effects.

## Material and Methods

### Plant material

*N. grandiflora* leaves were collected in the forest area belonging to Jaguari city, State of Rio Grande do Sul, Brazil at 29°26′25.09" S and 54°40′27.73" W. Access to the national genetic patrimony was given by the National Council of Scientific and Technological Development (CNPq, Brazil: #010191/2014-3). Leaves were gathered between October and November 2013 during plant flowering or fructification phenological stages. Botanical identification was performed by Solon Jonas Longhi and a voucher specimen was deposited at the Herbarium of the Biology Department of Federal University of Santa Maria, Santa Maria, RS, Brazil (SMDB 13.162).

### Essential oil (EO) extraction

EO was extracted 1 day after the plant material was gathered. We then applied hydrodistillation for 3 h using a Clevenger type apparatus, in triplicate (19). A 20 g leaf sample was dried in an oven at 40°C until it reached a constant weight, and it was then used for yield measurement (% w/w). The extracted EO was stored at -4°C in glass bottles in a light-free environment until chemical analysis and fractioning.

### Phytochemical analysis and DHF isolation

Both qualitative and quantitative analysis of the EO and DHF were performed using gas chromatography-mass spectrometry (GC-MS) and gas chromatography with a flame ionization detector (GS-FID). Detailed information of these methods can be found in Supplementary material 3. Isolation of DHF was conducted as stated in Supplementary material 4. In sequence, in the stationary phase three successive partition steps were performed in distinct chromatographic columns (CCs) using silica gel 60 (70-230 mesh, Macherey-Nagel, Germany). The fractions collected from the CCs were combined according to their thin layer chromatography (TLC) profile (Supplemental material 5). The substance recovered from the CCs was compared with an authentic DHF sample from our laboratory, which was identified by H^1^ and C^13^ nuclear magnetic resonance spectroscopy (Silva DT, Silva LL, Bianchini NH, Baldisserotto B, Longhi SJ, Heinzmann BM, unpublished data). The isolated DHF was kept in a light-free compartment at -4°C until chemical analysis and biological tests were performed.

### Drugs

Diazepam (DZP), a benzodiazepine drug with action on GABA_A_ receptors, was obtained from LCQ SM Pharmaceutical Enterprises, Brazil) and diluted in Tween 80 (0.02% in distilled water). Flumazenil (Flumazil¯, Cristália, Brazil) was incorporated into the water for the anesthesia/sedation reversion process. The DHF was diluted in ethanol 95% (1:10) before water incorporation. The vehicle group used in all experiments was composed of Tween (0.02% in distilled water), with ethanol at the highest concentration as a diluent.

### Animals

Silver catfish were purchased from a local fish culture and kept in continuously aerated 250-L tanks (induction experiments: 4.38±0.13 g juvenile fish/L; cortisol experiment: 12.62±1.06 g adult fish/L). The fish were kept under controlled water parameters suitable for the species, which are presented below as means±SE for all experiments. Dissolved oxygen (7.48±0.08 mg/L) and temperature (19.02±0.12°C) were determined using a YSI oxygen meter (Model Y5512, USA). For pH assessment (7.35±0.08), a DMPH-2 pH meter was used (Digimed, Brazil). Total ammonia levels (1.27±0.07 mg/L) were calculated using a salicylate method (20). A semi-static system with 50% daily water change was maintained to keep fish in a fresh environment. The subjects were fed once a day with commercial feed (28% crude protein) and fasted for 24 h prior to the experiments. Each animal was tested once and one at a time for all experiments performed in this study. The protocol was approved by the Ethical and Animal Welfare Committee of the Federal University of Santa Maria (Process #46/2010).

### DZP combination assay

An anesthetic and sedative induction protocol was conducted using fish juveniles (7.36±0.006 g, 9.07±0.004 cm; n=8) to evaluate potential positive interactions between DZP and DHF. Experimental aquaria (2L capacity) contained DZP (42.7 mg/L), DHF (5, 10, 15, 20, 25 or 50 mg/L) or a combination of the two. Water and vehicle control tests were carried out under the same conditions. DZP concentration was chosen based on previous work providing a standardized concentration of 150 µM DZP (corresponding to 42.7 mg/L) for fish sedation (4,21,22). The behavior protocol to evaluate the anesthetic/sedative induction and recovery was adapted from Gomes et al. (23). This approach describes six stages of progressive CNS depression: S1) light sedation, normal body movement with partial loss of reactions to external stimuli (tested by hitting a glass rod on the bottom of the aquarium); S2) deep sedation, animal rises to water surface and exhibits shaking behaviors (trembling/shaking body movements while moving toward the surface) and showing no reaction to external stimuli; S3a) partial loss of equilibrium, animal loses swimming posture, turns sideways but still swims; S3b) total loss of equilibrium, swimming skills cease, the animal lies at the bottom of the aquarium but still reacts to contact stimuli (tested by touching a glass rod on the caudal peduncle); S4) deep anesthesia, animal lies at the bottom of the aquarium and shows no reaction to contact stimuli; S5) medullar collapse (respiratory movement ceases). As S1 is difficult to accurately classify and can be misjudged, we chose not to measure it in this study. Consequently, the observation protocol comprised stages S2 to S4. Changes and reactions in animal behavior were observed and recorded by a trained analyst. Animals were kept in the aquarium until the anesthetic stage was reached or for 30 min. When an induction stage was reached by the fish, the corresponding time on a digital stopwatch (ZSD-808, Hong Kong) was recorded. After the induction protocol, the juveniles were moved to a drug-free aquarium to record the recovery time at which animals showed normal swimming behavior and responses to external stimuli. When recovered, fish were grouped according to protocol and relocated to a constantly aerated 40-L aquarium for observation of any abnormal behavior, diseases, or mortality over 48 h. This post experimental observation time protocol is well established in studies involving silver catfish (4,7,8,11,14,21,22).

### GABAergic pathway involvement

Heldwein et al. (14) outlined the evaluation in fish models of drugs interacting with the GABA_A_receptor. Firstly, fish juveniles (10.42±0.28 g, 9.99±0.13 cm; n=18, induction group; n=6, recovery group) were used to evaluate the reversal of depressant effects in a recovery bath containing flumazenil, a well-established benzodiazepine site antagonist of the GABAergic receptor subtype A (4,21,21 24). Animals were placed in aerated experimental aquaria (2 L capacity) with water only (1 L), water plus 42.7 mg/L DZP, water plus DHF at 20 (sedative concentration) and 50 mg/L (anesthetic concentration) or water plus a combination of DZP and DHF. Animals remained in the aquarium until anesthesia was achieved or for a maximum observation time of 30 min. Subsequently, fish were moved to aerated aquaria containing water and 1.5 or 3 mg/L flumazenil. Fish behavior was classified according to the following: 0=no movement; 0.5=caudal peduncle stimulus reaction; 1=movement but without posture; 1.5=static conduct after erratic swimming; 2=normal swimming without external stimulus reflex; 2.5=static conduct after normal swimming and no external stimulus reflex; 3=normal swimming with external stimulus reflex. The recovery performance was scored at 1, 5, 10, 15, and 20 min and then the scores were summed.

### Long-term exposure

To verify whether long-term exposure to DHF induces side effects, juveniles fish (8.12±0.04 g, 9.53±0.05 cm; n=6) exposed to sedative concentrations of DHF were observed for 24 h. Animal behavior was observed at 0.5 h after exposure and at 1, 2, 3, 4, 6, 8, 10, 12, and 24 h. The stage of CNS depression was recorded at these times. Water and vehicle controls were run simultaneously, and DHF was applied at concentrations of 2.5, 5, 10 and 20 mg/L. After 24 h, the animals were relocated to DHF-free aquaria for 48 h of surveillance for signs of toxicity and/or mortality.

### Stress response to DHF

Initially, adult fish (87.79±1.55 g, 25.18±1.75 cm) were grouped according to protocol in 250-L tanks with 36 animals in each. Fish were moved to experimental aquaria containing 1 L of water (1 fish per aquarium) and exposed to 20 or 50 mg/L DHF, water or a vehicle control until anesthesia was achieved or for a maximum observation time of 30 min. The stress protocol consisted of suspending the animal in air for 1 min following drug exposure. Aerial exposure is a previously described technique for stress response induction in silver catfish (8,25). The basal group consisted of unhandled fish, i.e., fish were moved from the 250-L tank directly to blood sampling. Animal blood was sampled after the stress protocol (time zero), after which the fish were moved to drug-free aquaria until sampling times at 5, 15, 30, 60 or 120 min. Six animals were used at each time and eight formed the basal group. Blood was collected only once from the caudal peduncle of each fish using heparinized syringes. It was then centrifuged at 3000 *g* for 15 min at 4°C and stored at -20°C until analysis. Subsequently, fish were euthanized by spinal cord splitting. Plasma cortisol concentrations were analyzed in duplicate using an enzyme-linked immunosorbent assay (ELISA) kit (Diagnostics Biochem Canada Inc., Canada). The absorbance was assessed in a microplate reader (Thermoplate, Brazil) at 450 nm. Inter- and intra-assay variation coefficients were 5.15±0.53 and 4.13±0.67%, respectively.

### Statistical analysis

Data are reported as means±SE. Initially, data were submitted to Levene’s test to determine homogeneity of variances. We performed two-way ANOVA followed by a *post hoc* Tukey’s test and one-way ANOVA followed by a *post hoc* Dunnett’s test, where appropriate. For non-parametric data, we employed Kruskal-Wallis ANOVA by ranks followed by Dunnett’s test, Mann-Whitney rank-sum test or Scheirer-Ray-Hare extension of the Kruskal-Wallis test followed by a *post hoc* Nemenyi test, as required.

## Results and Discussion

### EO chemical composition and DHF isolation

EO of *N. grandiflora* leaf (0.9454±0.01 g/mL) yield was 0.46±0.02%, representing an appropriate yield during the plant flowering stage (Silva DT, Silva LL, Bianchini NH, Baldisserotto B, Longhi SJ, Heinzmann BM, unpublished data). Detailed composition data is reported in Supplementary material 1. The major component of the EO was DHF, comprising 24.7% of its total content. The final column sample of DHF [α]D = + 172.231 (c 0.2456, CHCl_3_) amounted to 1.03 g, with 100% purity according to GC-FID analysis (Supplementary material 2). *N. grandiflora* is the only currently known (+)-DHF source as dextro-gyrate dehydrofukinone has not been extracted from any other species (17,16,26). The method of DHF isolation recovered 46.35% of the total contained in the EO sample subjected to column chromatography. Although the chemical synthesis of this sesquiterpene provides an overall yield reaction of 48-88%, as there is no synthesis route proposed for (+)-DHF isomers, the isolation process of EO is a suitable alternative to obtain this compound (16).

### DZP and DHF combination assay

**Figure 1:**
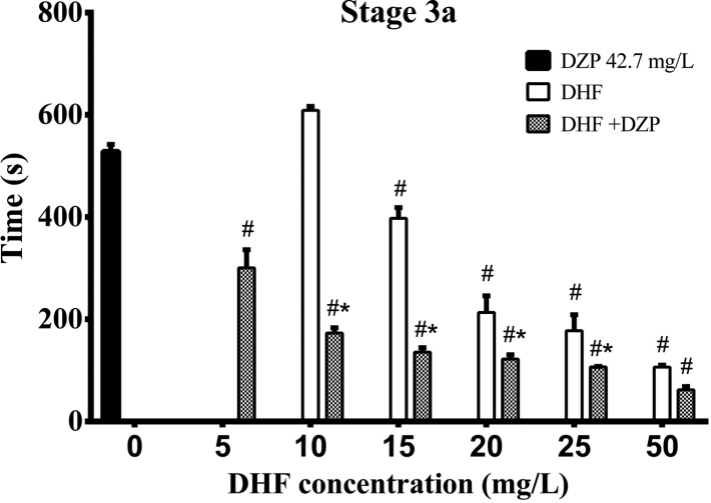
Effect of dehydrofukinone (DHF) and diazepam (DZP) or their combination in silver catfish at stage 3a of central nervous system depression. Data are reported as means±SE. Latency to reach stage 3a (partial loss of equilibrium, animal loses swimming posture, turns sideways but still swims) is reported in seconds. *P<0.001, DHF *vs* DHF+DZP at the same concentration (two-way ANOVA, *post hoc* Tukey’s test). ^#^P<0.001 compared to 42.7 mg/L DZP group (Kruskal-Wallis ANOVA by ranks, with *post hoc* Dunnett’s test).

Regression analysis showed a concentration-response relationship for DHF in S3a and recovery time. This relationship was also seen following exposure to a combination of DHF and DZP in all pre-anesthetic stages (Table 1). All DHF concentrations induced sedation (S2) in fish more quickly than DZP. DZP combined with the lowest DHF concentration (5 mg/L) did not diminish induction time. However, the presence of DZP postponed S2 induction with a DHF concentration of 10 mg/L. In concentrations above 15 mg/L, the DHF and DZP combination decreased the time taken to achieve S2. The next step of CNS depression, S3a, was achieved with the same speed by animals exposed to DZP or DHF at 10 mg/L. DHF at 5 mg/L did not induce this stage. However, when combined with DZP this degree of sedation was reached faster than with DZP alone. This positive interaction between DHF and DZP was detected at all combined concentrations and S3a was reached progressively faster with increased DHF concentration (Figure 1). DHF showed four times the potency of DZP, since no significant difference was observed between DHF 10 mg/L and DZP 42.7 mg/L at stage 3a. To reach this sedation level, the presence of DZP is inessential when 50 mg/L DHF is applied.

**Table:**
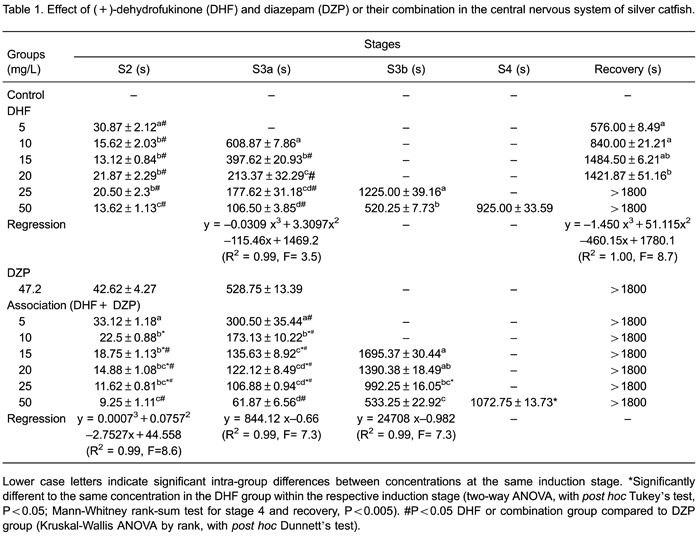

Whilst DZP did not induce S3b or anesthesia, it produced a depressed state in the fish that was not reversed in the drug-free aquarium within the observation period. This was true in combination with DHF at all concentrations. We believe that this interaction occurred due to presence of DZP, because fish subjected to DHF alone at sedative concentrations lower than 25 mg/L recovered their capacities during the observed time (30 min). However, those subjected to DHF at 25 and 50 mg/L did not return to normal behavior within the 30 min recovery session. The sustained depressant effect at higher DHF concentrations, even in a drug-free aquarium, is unsurprising due to the lipophilic character of DHF. The log P (partition-coefficient into water/octanol mixture) of DHF is about 4.0483, which is within the range of lipophilic drugs (27). During the depression of the fish CNS, high log P drugs are likely to accumulate in fat tissue, lingering there until a complete clearance process occurs (11). This slower dissipation of depressant drugs has been observed elsewhere in silver catfish juveniles exposed to 20-190 mg/L of the sesquiterpenoid globulol (21).

DHF at 25 mg/L combined with DZP induced S3b sooner than the same concentration of DHF alone. DZP and DHF alone (15 and 20 mg/L) were ineffective in inducing S3b. These concentrations when co-applied induced S3b in the fish, indicating once more a positive interaction between DZP and DHF. It is worth noting that the presence of DHF in the combination group decreased latency to reach all pre-anesthetic stages compared with the DZP alone group. Two drugs are considered to have independent action when the presence of one does not affect the receptor binding of the other, otherwise there will be an interaction between them (28,29). Considering that both drugs induced sedation, but that sedation induction times were decreased in the combination group compared with DZP alone, we infer the presence of a synergic or additive effect between DHF and DZP. Tallarida (28) assumes that synergic or additive effects between two drugs can aid prediction of the mechanism of action. This idea has been confirmed in some studies involving DZP and EO. For instance, in a model using silver catfish juveniles, the EO *Ocimum gratissimum* induced anesthesia in an interactive way when co-applied with 150 µM DZP. In addition, this EO showed depression of the fish CNS via a mechanism involving GABAergic transmission (22).

Conversely, S3b induction with DHF at 50 mg/L did not require DZP for a faster effect. Anesthetic stage S4 was reached only in fish exposed to DHF at 50 mg/L alone or in combination with DZP. Indeed, the presence of DZP increased the latency required to reach anesthesia compared with 50 mg/L DHF alone. Therefore, we would hypothesize that DZP, which has high affinity for the benzodiazepine site on GABA_A_ receptors, competes with DHF for the binding site. However, specific binding assays need to be performed to test this hypothesis.

### GABAergic pathway involvement

**Figure 2:**
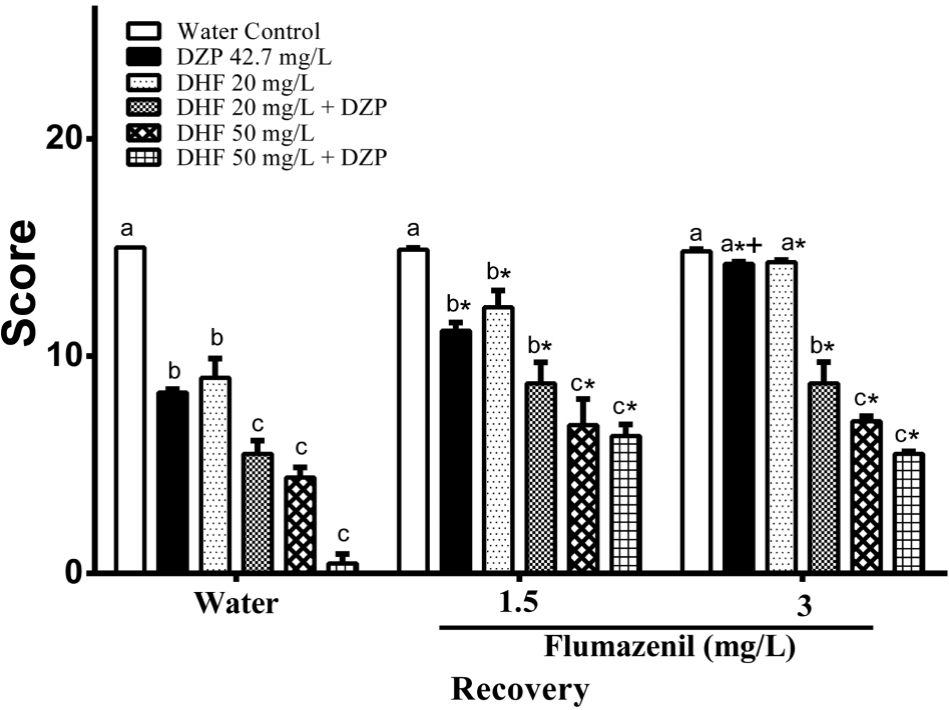
Anesthesia and sedation reversal of dehydrofukinone (DHF), diazepam (DZP) or their combination, in water or with GABA_A_ receptor benzodiazepine site antagonist, flumazenil. Data are reported as means±SE. Lower case letters indicate significant differences between treatments within the same recovery bath (P<0.001). *Significantly different compared to the respective treatment in water recovery bath (P<0.005). ^+^Significantly different compared to the respective treatment in 1.5 mg/L flumazenil recovery bath (P<0.05). Scheirer-Ray-Hare extension of the Kruskal-Wallis test was performed with *post hoc* Nemenyi test.

To elucidate whether the GABAergic system is implicated in depressant effects of DHF on the CNS, we investigated the involvement of GABA_A_ receptors in DHF-induced anesthesia and sedation in fish. For this purpose, anesthetized or sedated fish were transferred to flumazenil baths. Flumazenil, an imidazobenzodiazepine, blocks central effects of benzodiazepines by competitive interaction at the GABA_A_ benzodiazepine receptor site. This drug has a short half-life, estimated to be around 57 min in monkeys (30). Therefore, it is often used as a reversal agent for benzodiazepine-induced sedation and a treatment for benzodiazepine dependence (31). Earlier studies reported 5 µM (corresponding to 1.5 mg/L) flumazenil was a suitable concentration for reversal of DZP-induced CNS depression in fish (14,21,22). In this study, we raised the concentration of flumazenil to 3 mg/L to ensure total reversal of fish CNS depression, as full recovery is not seen with a bath of 1.5 mg/L flumazenil. Consistent with previous findings (17,21,22), flumazenil did not affect normal fish behavior at any concentration (P>0.05 *vs* water bath recovery). Vehicle and water controls were carried out alongside all recovery groups and did not differ statistically (P=0.999; data not shown). As expected, DZP sedated fish had higher recovery scores when exposed to 1.5 mg/L flumazenil compared with recovery in a water bath and showed total recovery when exposed to 3 mg/L flumazenil, validating the applied method (Figure 2).

Animals pretreated with a sedative concentration of DHF (20 mg/L) had higher recovery scores in a 1.5 mg/L flumazenil bath compared with a water recovery bath. The same group exposed to a 3 mg/L flumazenil bath returned to normal behavior after 20 min. They therefore showed the same behavior as DZP sedated fish. However, fish sedated with 20 mg/L DHF scored higher than those exposed to 20 mg/L DHF in combination with DZP when recovering in a water bath. Reversal of the sedative state using 1.5 mg/L flumazenil brings the recovery scores of these two treatments to the same level. Meanwhile, a 3 mg/L flumazenil bath did not bring fish pre-treated with a combination of DZP and DHF back to normal performance. Flumazenil interrupted the action of 20 mg/L DHF in a concentration-dependent manner. However, the fish treated with 20 mg/L DHF in combination with DZP had higher recovery scores in a 1.5 mg/L flumazenil bath compared with water recovery, but not when compared with 3 mg/L flumazenil. Thus, the presence of DZP impaired the reversal action of flumazenil on DHF-induced sedation.

DHF at 50 mg/L and DHF at 20 mg/L in combination with DZP achieved the same recovery score level after 20 min of water bath. This was not seen when fish were subjected to either a 1.5 or 3 mg/L flumazenil bath. The presence of DZP with 50 mg/L DHF pretreatment did not affect the recovery from anesthesia. Groups recovering in 1.5 or 3 mg/L flumazenil baths both had higher scores than recovery in a water bath. Although the score obtained at 3 mg/L flumazenil did not differ from that acquired at 1.5 mg/L flumazenil, these findings demonstrate that antagonist concentrations used in this study entirely reversed DHF sedation but not DHF anesthetic effects in fish. Natural compounds, specifically some monoterpenoids, modulate GABAergic function (7,20). Furthermore, other neuronal pathways, such as the serotoninergic system, may play a role in depressant effects of EO-derived compounds on the CNS (5,32). Taking these results together, we believe that DHF action involves GABAergic transmission via the benzodiazepine site at the GABA_A_ receptor. Nevertheless, since the effect of an anesthetic concentration of DHF was not entirely reversed by the specific benzodiazepine site antagonist, other sites or even other receptors may be involved (Figure 2).

### Long-term exposure

**Figure 3:**
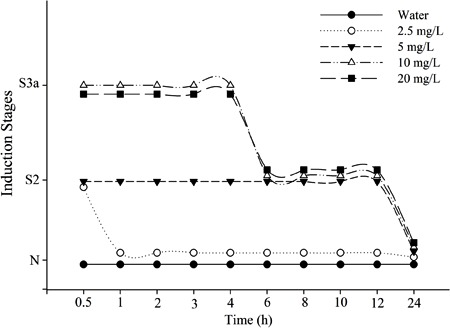
Qualitative observations of long-term exposure to dehydrofukinone (DHF) at sub-anesthetic concentrations. The time that fish remained in the stage is reported in hours on the x-axis. N indicates normal behavior, S2 indicates light sedation, and S3a indicates partial loss of equilibrium.

As a standard practice, fish are usually exposed to anesthetic just long enough to achieve anesthesia (30 min) (4,7,8,11,14,21,22,24). With this exposure time, no side effects are reported for DHF at concentrations used in this study (18). However, sedative concentrations of anesthetic drugs are often employed during transport of fish, and fish behavior following extended contact with DHF (1-24 h) have not been previously evaluated (11). Therefore, we performed a set of long-term sedative baths, to investigate potential side effects. Extended exposure to anesthetic concentrations can lead fish to enter S5 of CNS depression (medullar collapse). To avoid this, sedative concentrations were used. The stages of CNS depression observed in the fish across 24 h of DHF exposure are presented in Figure 3. Sedative effects of DHF treatment in fish have previously been reported above 9 mg/L (18). In this assay, fish exposed to 2.5 mg/L DHF achieved sedation stage 2 after 1 min (data not shown). S2 was maintained with DHF at 2.5 mg/L for 30 min and then normal behavior was resumed. Thus, depressant effects of DHF occur even at concentration as low as 2.5 mg/L when compared to other concentrations of fish sedatives reported in literature (4,7,8,14,21,22,23).

Fish sedation with other EO derivatives is also described in the literature. The sesquiterpenoid globulol and the monoterpenoid 1-terpinen-4-ol sedated silver catfish juveniles at 10 and 3 mg/L, respectively. However, sedation induced by 1-terpinen-4-ol was not as long lasting as DHF, which provides better maintenance of sedation at lower concentrations (21). Both 10 and 20 mg/L DHF induced S3a depression continuously for up to 4 h. Additionally, after 6 h exposed to 10 or 20 mg/L DHF, sedation lightened from S3a to S2, and fish remained at this stage for 12 h. After 24 h, animals exposed to any DHF concentration showed normal behavior. At 48-h post-assay, toxicity signs, such as mucus loss, disease, or mortality, were not detected. This suggests superiority of DHF at sedative concentrations compared with conventional anesthetics. Although the widely studied fish anesthetic tricaine methane-sulfonate (MS-222) provides sedation in several fish species at concentrations higher than 20 mg/L (10), some synthetic anesthetics applied in aquaculture can cause mucus loss and retinal and gill damage (11).

Altogether, our results suggest that DHF can be used for prolonged maintenance of sedation under apparently safe conditions. However, more studies investigating toxicological traits should be performed to justify DHF pharmacological safety.

### Stress response to DHF

**Figure 4:**
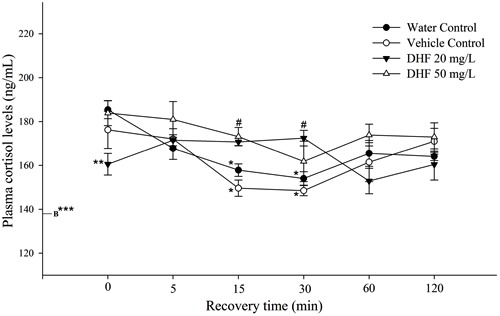
Plasma cortisol response to dehydrofukinone (DHF) exposure. Data are reported as means±SE. B denotes cortisol basal value. ^#^P<0.05 compared to vehicle control group at the same time. *P<0.05 compared to time zero in the same group (two-way ANOVA, with *post hoc* Tukey’s test). **P<0.05 compared to water control group at the same time. ***P<0.05 compared to all groups at all times (one-way ANOVA, with *post hoc*Dunnett’s test).

As described in the literature, fish are very sensitive to their environment. The perception of any signs of danger, oxygen-poor water, light changes, or even the presence of chemical agents may trigger stress in fish (11). Stress events activate neuroendocrine systems to release hormones, such as catecholamines and corticosteroids. Cortisol, a corticosteroid hormone, is gradually released, while catecholamines show transient action. Elevated plasma levels of cortisol delay the return to basal values, so its measurement is used to evaluate the stress response in fish (11,33). High fish stocking can also be stressful for the animals (34). In this study, we grouped fish in 250 L-tanks, consistent with densities deemed not to be stressful (35). The hormonal response of fish to sedative and anesthetic concentrations of DHF are presented in Figure 4. Baseline plasma cortisol concentrations were 137.93±0.77 ng/mL and cortisol levels in a water control condition diverged from baseline levels, validating the stress protocol. Animals exposed to a vehicle control bath behaved as fish under water control bath at all analyzed times (P=0.7).

Cortisol levels at 15 and 30 min were significantly lower than at time zero in both water and vehicle control groups. Cortisol tended to increase, 60 min after stress induction, but did not differ significantly from either 15 or 30 min. Our data showed a cortisol peak between 0 and 5 min after air exposure. At time zero after stress protocol, DHF at 20 mg/L decreased fish cortisol levels compared with the water control group. Therefore, a pre-exposure bath of 20 mg/L DHF decreased plasma concentrations of this hormone at the time where the cortisol peak was detected in the water control group. DHF at 50 and 20 mg/L induced higher cortisol plasma concentrations than the vehicle control group at 15 and 30 min after stress induction, respectively. However, no significant difference was observed compared with the water control group at these times. Furthermore, plasma cortisol measured in fish exposed to 50 mg/L DHF remained at the same level as water and vehicle groups at time zero (P<0.05; data not shown).

Positive modulation of GABA_A_ receptors promotes a downregulation in the hypothalamic-pituitary-adrenal axis, diminishing stress hormone release (36). In contrast, some GABA_A_ agonists promote an increase in stress hormonal response under stressful conditions (37). Here, DHF exposure did not increase fish cortisol levels above those of the water control group at any time point. Cunha et al. (8) reported that pretreatment with eugenol or the EO of *Lippia alba* did not change plasma cortisol levels in fish submitted to a stress protocol. However, other EO components, such as isoeugenol, decreased plasma cortisol levels in fish. The anesthetics and sedatives that decrease or do not modify plasma cortisol levels are recognized as relatively safe drugs for aquaculture practice (11). Cortisol response to drugs is currently used as a screening tool in aquatic species for anesthetics. DHF appears to favorably modulate this physiological stress pathway. These results are consistent with investigations of long-term exposure to DHF, suggesting its safety and applicability as a sedative/anesthetic in fish models.

In conclusion, DHF is effective as a sedative at a concentration 20-fold lower than that required for anesthesia in this fish model. This molecule operated in a collaborative way with DZP at sedative concentrations and GABA_A_receptors appear to be involved in its depressant action on the CNS. DHF induced no side effects even after 24-h exposure and at sedative concentrations it prevented stress-induced cortisol increase. Therefore, DHF proved to be a relatively safe sedative/anesthetic that interacts with GABAergic and cortisol pathways in fish.

## Supplementary Material


