# Supplementary material for: Effect of (+)-dehydrofukinone on GABAA receptors and stress response in fish model
Source: Braz J Med Biol Res. 2015 Nov 27;49(1):e4872. doi: 10.1590/1414-431X20154872 (PMC4681417; doi:10.1590/1414-431X20154872)

SUPPLEMENTARY MATERIAL

Essential oil (EO) and (+)-dehydrofukinone (DHF) identification by gas chromatography-mass spectrometry (GC-MS) and quantification by gas chromatography with a flame ionization detector (GS-FID) methods. Description of the DHF isolation process. Thin layer chromatography profile and procedure.

Supplementary material 1. EO composition of *Nectandra grandiflora* (Lauraceae) leaves.

Peak	RT	Components	RI_C	RI_L	%
1	10.32	α-Pinene	931	937^N	2.14
2	12.00	*β*-Pinene	973	978^N	1.27
3	14.61	(*Z*)-Ocimene	1039	1038^N	0.26
4	14.99	(*E*)-Ocimene	1049	1048^N	0.63
5	17.02	Linalool	1101	1101^N	3.42
6	20.27	(2*E*)-Hexenyl-butyrato	1188	$1191^{N/A}$	0.52
7	27.22	*β*-Elemene	1393	1394^N	0.47
8	28.08	Aromadendrene	1420	1418^N	1.05
9	28.78	NI	1443	–	2.75
10	29.65	*α*-Muurolene	1471	1472^N	1.04
11	29.88	Allo-Aromadendrene	1479	1478^N	1.29
12	29.99	Amorpha-4,7 (11)-Diene	1482	1479^A	3.78
13	30.20	Valencene	1489	1490^N	1.39
14	30.37	Eremophilene	1495	1489^N	0.90
15	30.48	Bicyclogermacrene	1498	1500^A	5.93
16	30.74	NI	1507	–	9.12
17	30.81	NI	1509	–	2.59
18	31.75	NI	1541	–	0.75
19	32.86	Spathulenol	1579	$1578^{N/A}$	3.18
20	33.05	NI	1586	–	0.75
21	33.18	NI	1590	–	0.57
22	33.36	NI	1596	–	0.58
23	34.94	NI	1653	–	7.47
24	35.05	Selin-11-en-4-α-ol	1657	1659^A	5.09
25	37.70	NI	1755	–	3.66
26	37.85	NI	1761	–	1.32
27	39.07	Dehydrofukinone	1808	*	24.70
28	39.39	NI	1820	–	6.19
29	42.18	Rumuene	1933	1930^N	1.62
30	44.71	(-)-Kaurene	2040	2041^N	5.49
Total percent identification					70.20

RT: column retention time; RI_C: calculated retention index; RI_L: literature retention index; N: NIST, 2008; A: Adams, 2009; NI= no identified compound. *Compared with an authentic DHF sample from our laboratory, which was identified by H^1 and C^{13} nuclear magnetic resonance spectroscopy (Silva DT, Silva LL, Bianchini NH, Baldisserotto B, Longhi SJ, Heinzmann BM, unpublished data).

1. National Institute of Standards and Technology: NIST 2008. *Mass spectral library (NIST/EPA/NIH),* Gaithersburg, USA, 2008.
2. Adams RP. *Identification of Essential Oil Components by Gas Chromatography/Quadrupole Mass Spectroscopy*. Allured Publishing Corporation. Illinois, 2009.

Supplementary material 2. ***N. grandiflora*** **EO (black line) and isolated (+)-dehydrofukinone (red line) FID chromatograms.**

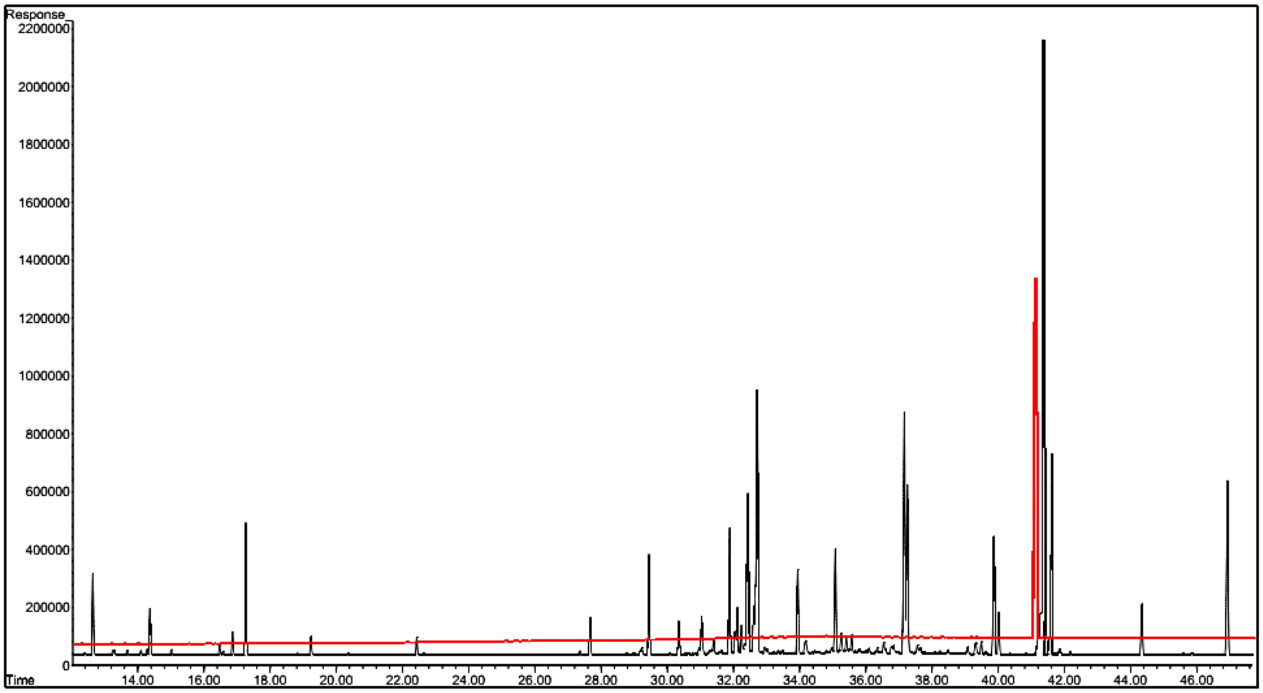

Supplementary material 3. Phytochemical analysis.

GC-MS was executed in Agilent 6890A gas chromatography coupled with a 5973 mass selective detector using a non-polar HP5-MS fused silica capillary column (5% phenyl, 95% methylsiloxane, 30 m x 0.25 mm i.d. x 0.25 µm film thickness) and electron ionization mode at 70 eV. Helium was used as carrier gas in a flow rate of 1.0 mL min^{-1}, injector temperature set at 250°C and detector at 280°C. Oven temperature kept at 40°C for 4 min was raised to 320°C at a rate of 4°C per min. Sample solutions of 1 µL (2:1000 in hexane, v/v) were injected in split inlet mode (1:100 ratio). Kovats retention indices were calculated using a homologous series of C7-C31 n-alkanes injected under the same conditions of the samples. The EO constituents were identified by mass spectra and Kovats retention index comparison with data from the National Institute of Standards and Technology Mass Spectral Library (1) and with the literature (2). FID analysis was run using a column with equivalent characteristics and the same oven parameters than GC-MS. Injection and detection temperatures were programmed at 300°C and the split inlet mode ratio was 1:50. EO compounds relative percent was estimated by under peak area integration obtained from FID chromatograms.

1. National Institute of Standards and Technology: NIST 2008. *Mass spectral library (NIST/EPA/NIH),* Gaithersburg, USA, 2008.
2. Adams RP. *Identification of Essential Oil Components by Gas Chromatography/Quadrupole Mass Spectroscopy*. Allured Publishing Corporation. Illinois, 2009.

Supplementary material 4. DHF Isolation process description

Column fractions were concentrated under reduced pressure, at 40°C for gas chromatography subsequential analysis. Proportion between sample and stationary phase was set at 1:100 in all separation steps. The first column chromatography (CC) (650 mm length x 75 mm diameter; eluent: hexane/acetone, 95:5; flash flux; 100 mL/ fraction) comprises crude EO (9 g; 24.7% DHF) separation into 7 fractions. The third fraction (4.3g; 55.03% DHF) was separated in another CC (650 mm length x 45 mm diameter; flux: 0.8 mL/min; 25 mL/fraction) eluted with hexane/ether (90:10) in two repetitions. Once more, the third fraction from each replicate (1.306 g; 82.6% DHF) was partitioned in CC (640 mm length x 23 mm diameter; flux: 0.5 mL/min; 10 mL/fraction) eluted with hexane/acetone (97:3). This last step employed $AgNO_3$ (polarity enhancer) saturated silica gel (1:4) as stationary phase (1). Silica gel was impregnated in 250 mL $AgNO_3$ solution (10% in water) and then submitted to rotaevaporator apparatus for solvent elimination.

1. Williams CM, Mander LN. Chromatography with silver nitrate. *Tetrahedron* 2001; 57: 425–447.

Supplementary material 5. Thin layer chromatography (TLC).

TLC analyses (aluminum sheets pre-coated with silica gel 60 F_{254} chromatoplates, Macherey-Nagel, Germany) were carried using the respective column eluent system and vanillin sulfuric acid/ UV 365 nm detection.

TLC profile of *N. grandiflora* EO and isolated (+)-DHF under UV detection

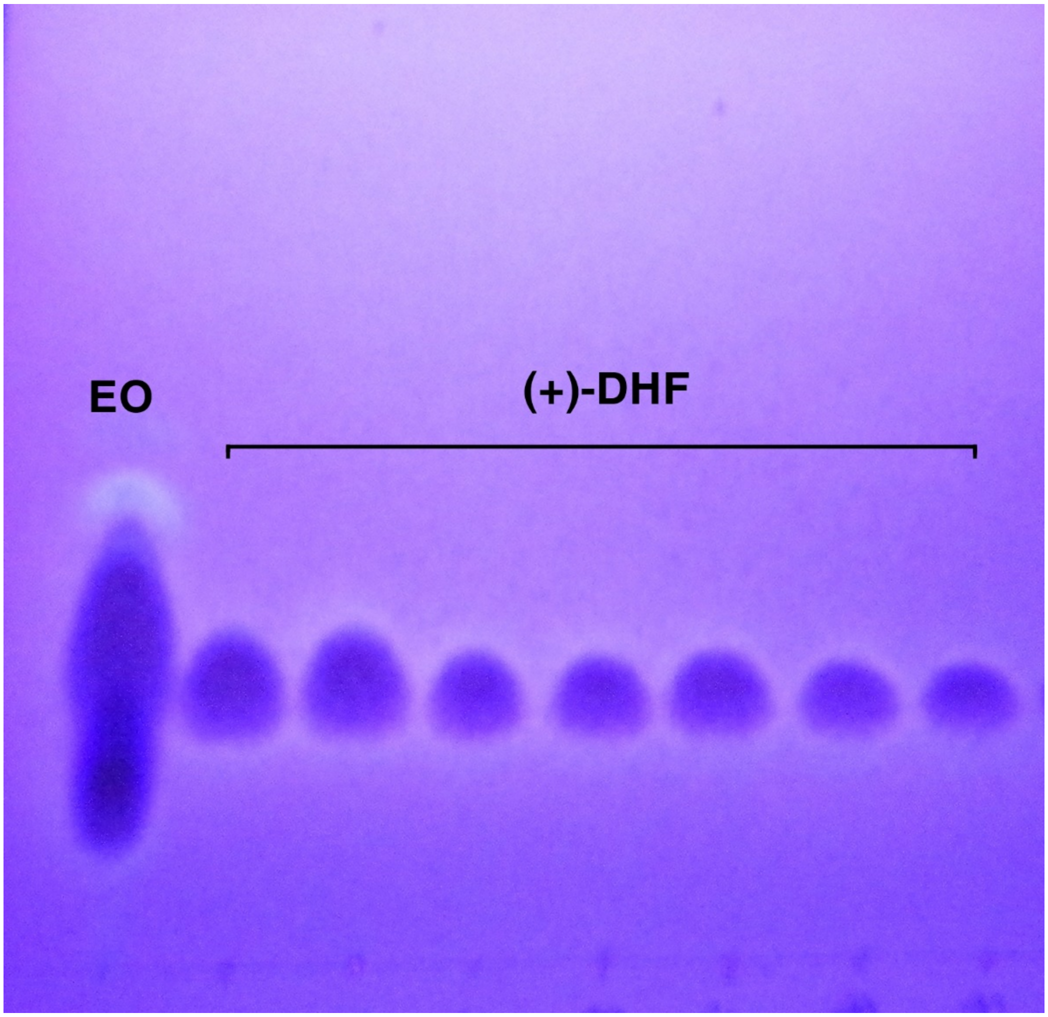

Supplement: Supplementary file 1 [file 1414-431X-bjmbr-1414-431X20154872-supp4872.pdf]
